# Inflammation-associated miR-155 activates differentiation of muscular satellite cells

**DOI:** 10.1371/journal.pone.0204860

**Published:** 2018-10-01

**Authors:** Yuta Onodera, Takeshi Teramura, Toshiyuki Takehara, Maki Itokazu, Tatsufumi Mori, Kanji Fukuda

**Affiliations:** 1 Division of Cell Biology for Regenerative Medicine, Institute of Advanced Clinical Medicine, Kindai University Faculty of Medicine, Osaka, Japan; 2 Department of Rehabilitation Medicine, Kindai University Faculty of Medicine, Osaka, Japan; 3 Kindai University Life Science Research Institute, Kindai University, Osaka, Japan; University of Minnesota Medical Center, UNITED STATES

## Abstract

Tissue renewal and muscle regeneration largely rely on the proliferation and differentiation of muscle stem cells called muscular satellite cells (MuSCs). MuSCs are normally quiescent, but they are activated in response to various stimuli, such as inflammation. Activated MuSCs proliferate, migrate, differentiate, and fuse to form multinucleate myofibers. Meanwhile, inappropriate cues for MuSC activation induce premature differentiation and bring about stem cell loss. Recent studies revealed that stem cell regulation is disrupted in various aged tissues. We found that the expression of microRNA (miR)-155, which is an inflammation-associated miR, is upregulated in MuSCs of aged muscles, and this upregulation activates the differentiation process through suppression of C/ebpβ, which is an important molecule for maintaining MuSC self-renewal. We also found that Notch1 considerably repressed miR-155 expression, and loss of Notch1 induced miR-155 overexpression. Our findings suggest that miR-155 can act as an activator of muscular differentiation and might be responsible for accelerating aging-associated premature differentiation of MuSCs.

## Introduction

Normal tissue renewal and regeneration mainly depend on the quality of tissue-resident stem cells. Muscle satellite cells (MuSCs) are myogenic stem cells required for regeneration of adult skeletal muscles. In response to injury or growth factor stimulation, MuSCs are activated and they proliferate. Following proliferation, the majority of MuSCs undergo myogenic terminal differentiation and perform *de novo* myotube formation, or fuse with damaged myofibers to repair the injury [1, 2]. Although transient and appropriately tuned activation is required for sustaining muscle repair, chronic or excessive inflammation can be deleterious, resulting in uncontrolled balance of self-renewal /differentiation, and finally triggering muscle wastage [3].

Aging contributes to degeneration of various tissues, including muscles. Age-related muscle wasting is characterized by the loss of muscle quantity and quality, and as well as declining numbers of MuSC [4–6]. Since it is a critical reason for stem cell deterioration in aged tissues, the altered expression of important signaling molecules has been reported to induce inappropriate stem cell activation and reduction of the stem cell pool. For example, age-related decreases in the expression of Notch signaling molecules has been found in muscles [7, 8]. Interestingly, enhanced expression of myogenic genes such as *MyoD* and *Myogenin* have been found in aged muscles, suggesting committed status of the MuSCs [4, 9, 10]. Although the causes of muscular tissue atrophy during aging are still unclear, premature-activation of tissue stem cells could be an important cause of irreversible tissue deterioration. Barnet et al. suggested that elevated pp38, likely stimulated by the aged environment with increased cellular stress and inflammatory responses, prevents asymmetric p38MAPK signal transduction and generates lineage-committed daughter cells from MuSCs [11]. Recently, Rozo *et al*. found that decreasing β1-integrin levels in MuSCs are responsible for the aged phenotype, including reduced proliferation and a bias toward differentiation resulting in MuSC loss and impaired regeneration [12]. Clearly, the increased production of pro-inflammatory cytokines such as TNFα, IL-1β, IL-6, and IFNγ, which is a common feature of aging and chronic inflammation-associated diseases, becomes an important factor that induces the loss of muscle mass [3, 13, 14]. However, a complete understanding of the identities and roles of all molecular players involved in triggering abnormal stem cell activation in muscles remains elusive.

In recent decades, various roles of microRNAs (miRNAs) have been identified. miRNAs are a class of around 22-nucleotide-long noncoding RNAs that regulate gene expression at the post-transcriptional level [15–17]. miR-155 is a dominant inflammation-associated miRNA and is involved in stem cell differentiation and deterioration [18]. Interestingly, miR-155 is significantly upregulated in various human muscle disorders [19]. Furthermore, in aged tissues, upregulation of miR-155 has been reported [20–22]. However, its expression status in MuSCs of aged muscles, role in muscular homeostasis, and relationship with aging-associated muscle wasting are not understood.

We aimed here to clarify the above issues, and we found that miR-155 expression was highly upregulated in the MuSCs of senile muscles. Furthermore, we demonstrated that miR-155 is involved in activating differentiation through suppressing CCAAT/enhancer binding protein beta (C/ebpβ) expression, and that upregulation of miR-155 was partly induced by reduced Notch1 expression.

## Materials and methods

### Antibodies and primers

Antibodies and dilution conditions are presented in Table 1. Primers for quantitative PCR are described in Table 2.

**Table 1:** Antibodies used in the present study.

Antibody	Company	Application	Dilution
CD45-FITC (30-F11, 35–0451)	TONBO	FACS	1:100
CD11b-FITC (M1/70, 35–0112)	TONBO	FACS	1:100
CD29-PE (HMß1-1, K0046-5)	MBL	FACS	1:100
CD31-FITC (390, 130-102-970)	Miltenyi Biotec	FACS	1:100
Integrin α7-APC (3C12, 130-103-356)	Miltenyi Biotec	FACS	1:100
Myogenin (F5D, 14-5643-82)	eBioscience	WB	1/ 1,000 in Immuno-enhancer
MyoD (5.8A, MA5-12902)	Invitrogen	WB	1/ 1,000 in Immuno-enhancer
Notch1 (D1E11, #3608)	Cell Signaling	WB	1/ 1,000 in Immuno-enhancer
GAPDH (sc-25778)	SCB	WB	1/ 5,000 in 0.2% Tween-TBS containing 10% Block-ace
Myosin IIb (D8H8, #8842)	Cell Signaling	WB	1/ 1,000 in Immuno-enhancer

**Table 2:** Primer sequences for quantitative RT-PCR, ChIP-PCR, and genotyping in the present study.

		Forward	Reverse
**qPCR**	***MyoD***	tccaactgctctgatggcatg	tcactgtagtaggcggtgtcg
	***Myogenin***	agcgcgatctccgctacagag	gaccgaactccagtgcattg
	***Mrf4***	agcaagagaagatgcaggagc	cttccttagcagttatcacgagg
	***Myf5***	aggtggagaactattacagcctg	ttcgggaccagacagggctgttac
	***Pax7***	tcgggttctgattccac	aaagccaaacacagcatc
	***C/ebpβ***	aagaagacggtggacaagctg	tgctccaccttcttctgcagc
	***Gapdh***	gtgaaggtcggagtgaacg	taaaagcagccctggtgac
**ChIP**	**miR155 Rbpj BR1**	tcacagactttcctcatgaaac	tcgtggcttggaaatttc
	**miR155 Rbpj BR2**	aatatagcccaagctgacc	tctactacatgaaaagcctagg
**Genotyping**	**miR155 Knockout**	tctgacatctacgttcatcc	actaactgtgtgcgtacacac

### Ethics statement

All procedures involving animals were approved by the Institutional Animal Care and Use Committee at Kindai University and were performed in accordance with the institutional guidelines and regulations. All surgery was performed under isoflurane anesthesia, and all efforts were made to minimize suffering. At the end of the studies, mice were sacrificed by cervical dislocation.

### MuSC collection from mice muscles

For this study we used 3-week old C57BL/6N (B6) male mice as young model and 1.5-year old B6 male mice as aged model (both are purchased from CLEA Japan Inc., Tokyo, Japan). Mice were euthanized and posterior biceps femoris (PBF) muscles were collected. To collect cell populations including SCs, the muscles were cut into small pieces with a scalpel, washed twice and digested with collagenase type I (Wako, Tokyo, Japan) for 15 min. The dissociated tissues were then washed twice with PBS (-) and incubated with anti-CD11b, -CD29, -CD31, -CD45, and -Integrin α7 (ITGA7) for isolating the muscle progenitor/ stem cell population. As a negative control, cells were incubated with isotype IgGs. CD11b^negative(neg)^/ CD31^negative^/ CD45^negative^/Integrin α7^positive^/CD29^positive^ cells were sorted using FACS Aria II (BD Biosciences).

### Quantitiative RT-PCR (qPCR) analysis for *mmu-miR-155-5p*

For miRNA qRT-PCR, total RNA prepared as above was reverse-transcribed using the Universal cDNA synthesis Kit II (Exiqon, Inc., Vedbaek, Denmark). The resulting cDNA was diluted 1:50 for qPCR. PCR was performed using an ExiLENT SYBR Green master mix (Exiqon) with the following miRCURY LNA PCR primer sets (Exiqon): *mmu-miR-155-5p* (ID 205930) and *U6 snRNA* (ID 203907). To obtain relative expression, the Ct (threshold cycle) values of miR-155 were normalized to the expression of U6 (ΔCt = Ct _miR-155_ − Ct _U6_) and compared with a calibrator using the "ΔΔCt method" (ΔΔCt = ΔCt _sample_ − ΔCt _control_). Data were expressed as mean values ± SD of 3 experiments. Statistical significance was evaluated by Student’s *t*-test with JMP software version 10.0.0 (SAS Institute, Cary, NC, USA).

### qRT-PCR

Total RNA was extracted from specimens using TRI Reagent (Molecular Research Center Inc., Cincinnati, OH, USA) and reverse-transcribed with the PrimeScript RT Master Mix Kit (Takara Bio Inc., Shiga, Japan). Quantitative real-time PCR with total cDNA was performed using Perfect real-time SYBR green II (Takara). PCR amplifications were performed on the Thermal Cycler Dice Real Time System Single at 95°C for 20 s followed by 40 cycles at 95°C for 5 s and 60°C for 30 s. To quantify the relative expression of each gene, the Ct (threshold cycle) values were normalized to that of *Gapdh* and compared with a calibrator using the ΔΔCt method (ΔΔCt = ΔCt _sample_ − ΔCt _control_). To prevent amplification of contaminating genomic DNA, we designed all primers to span at least one intron. Statistical significance was evaluated by Student’s *t*-test using JMP software version 10.0.0 (SAS Institute, Cary, NC, USA). Primer sequences are listed in Table 1.

### Western blot (WB) analysis

Cells were homogenized in SDS buffer and centrifuged at 9,000 × *g* for 10 min at 4°C to remove debris. Aliquots were subjected to polyacrylamide gel electrophoresis followed by electrotransfer onto a PVDF membrane (Hybond-P; GE Healthcare Japan, Tokyo, Japan). The blotted membranes were blocked overnight with Block Ace (Dainippon Sumitomo Pharma, Osaka, Japan) and then probed overnight with primary antibodies at 4°C. Detection was performed with horseradish peroxidase (HRP)-conjugated secondary antibodies and Immunostar LD (Wako) detection reagents. Antibodies are listed in Table 2.

### Cell culture and overexpression of *mmu-miR-155*

The mouse myoblast cell line C2C12 was cultured in DMEM (Wako, Tokyo, Japan) supplemented with 200 mM L-glutamine and 10% fetal bovine serum (Hyclone, Logan, UT, USA), in 5% CO_2_ at 37°C. For overexpression of miR-155, C2C12 cells at 80% confluency were transfected with miExpress EGFP-mmu-miR-155 plasmid (GeneCopoeia Inc. Rockville, MD, USA) or pPBQM-*mmu-miR-155* plasmid (a gift from Dr. Martin Lotz) using ScreenFect A (Wako). We also used a scrambled control sequence expression plasmid (CmiR0001-MR04, GeneCopoeia, Inc.) and a *mmu-miR-155* precursor expression plasmid (MmiR3427-MR04, GeneCopoeia, Inc.). The cumate-gene switch was activated by adding 30 μg/mL cumate (QM100A-1, System Bioscience Inc., Palo Alto, CA, USA). Myogenic differentiation was induced by culturing confluent C2C12 cells in DMEM containing 2% horse serum (Biowest USA, NW, USA) for 12 days.

### Muscle injury models and fluorescence activated cell sorting (FACS) of stem- progenitor cell populations

Muscle injury models were generated in 8-week old C57BL/6N (B6) male mice (CLEA Japan Inc., Tokyo, Japan). The mice were anesthetized by 2% isoflurane. Following anesthesia, the PBF muscles were aseptically exposed and three slits of 2 mm (width) × 3 mm (depth) were made using a scalpel. After 1, 3, 6, 9, and 28 days, the mice were euthanized and the PBF muscles were collected. To collect cell populations including SCs and progenitors using FACS, the muscle tissues were cut into small pieces with a scalpel, washed twice and digested with collagenase type I (Wako, Tokyo, Japan) for 15 min. The dissociated tissues were then washed twice with PBS (-) and incubated with anti-CD31 (FITC conjugated, 130-102-970, Miltenyi Biotec K.K., Tokyo, Japan), anti-Intα7 (APC conjugated, 130-103-355, Miltenyi Biotec K.K.), and anti-CD29 (PE conjugated, 130-102-994, Miltenyi Biotec K.K.) for isolating the muscle progenitor/ stem cell population. As negative controls, cells were incubated with PE- or FITC-conjugated isotype IgGs (Miltenyi Biotec K.K.). CD31^negative^/Integrin α7^positive^/CD29^positive^ cells were sorted using FACS Aria II (BD Biosciences).

### Generation of *mmu-miR-155* KO C2C12 cells

To generate a mmu-miR-155 knockout C2C12 cell line, we targeted the coding region of miR-155 using two guide RNAs: guide 1, GTGGAACAAATGGCCACCGT and guide 2, GTTGCATATCCCTTATCCTC. For the CRISPR-Cas9 treatment, C2C12 cells were dissociated with TrypLE Express, washed twice with Opti-MEM, and resuspended in 100 μL of Opti-MEM containing 7.5 μg of MLM3636 guide RNA expression plasmids (Addgene #43860) and 5 μg Cas9 expression plasmid pSpCas9(BB)-2A-Puro (PX459) V2.0 (Addgene #62988). After selection with 1 μg/mL puromycin for 2 days, the resulting transgenic C2C12 cells were seeded on 96-well plates at 1 cell per well in 10% FCS-DMEM using a BD FACS Aria II (BD Bioscience). For the genomic PCR for genotyping, C2C12 cells were lysed with KAPA Express Extract (Nippon Genetics Co, Ltd., Tokyo, Japan) and diluted in Tris-EDTA. Genomic PCR was performed using KOD FX Neo DNA polymerase (Toyobo Co. Ltd., Osaka, Japan) with genotyping primers listed in Table 2.

### Transplantation of C2C12 cells

Prior to transplantation, C2C12 cells were labeled with GFP using a piggybac-CAG-GFP plasmid (kindly gifted by Dr. Hitoshi Niwa, Institute of Molecular Embryology and Genetics, Kumamoto University). The cells were surgically transplanted in 8-week-old C3H male mice (SLC Japan Inc., Shizuoka, Japan) subjected to muscle injury as described above; the mice were subsequently injected into the slits with 1 × 10^5^ C2C12 cells in 2 μL of PBS. After 1, 3, 6, 9, or 28 days, the mice were euthanized, the PBF muscles were collected, and GFP positive cells were sorted by FACS for further analysis.

### Primary culture from muscular tissues and induction of Notch1 floxing

Notch1^flox/flox^ (JAX stock #007181) mice were obtained from Jackson laboratory (Bar Harbor, ME, USA). Primary cultures of MuSCs and myoblasts were performed following previously established methods with few modifications. Collagenase treated cells were harvested on iMatrix-511 (Nippi, Inc., Tokyo, Japan) coated dishes and cultured in 20% FCS-DMEM supplemented with 10 ng/mL bFGF for 1 week. Then the cells were transfected with 5 μg Cre-GFP plasmid (Addgene, #49054) with Lipofectamine 3000. After 24 hours of the transfection, GFP positive cells were sorted with FACS.

### Chromatin immunoprecipitation (ChIP) and qPCR

C2C12 cells were transfected with 5 μg Notch1-ICD domain expressing plasmid (Addgene, #26891). After 48 hours of the transfection, the cells were fixed with methanol-free formaldehyde and used for IP following the previously published method [20] with anti-Notch1 rabbit monoclonal antibody (D1E11, Cell Signaling Technology, Beverly, MA, USA). Putative Notch1 binding regions for ChIP-qPCR were identified using open data sets with ChIP-Atlas analyzer. ChIP-qPCR was performed with Perfect real-time SYBR green II (Takara).

### Statistical analysis

Significant differences were detected by Tukey-Kramer’s HSD test or Student’s *t*-test, as appropriate. P values less than 0.05 were considered significant.

## Results

### MuSCs of aged mice exhibited a differentiating phenotype

MuSCs were identified as CD11b^negative(neg)^/CD 31^neg^/CD45^neg^ and Integrin α7 (ITGA7)^positive(pos)^/CD29^pos^ population [23–25]. In this study, description of miR-155 means its dominant strand miR-155-5p.

**Fig 1:**
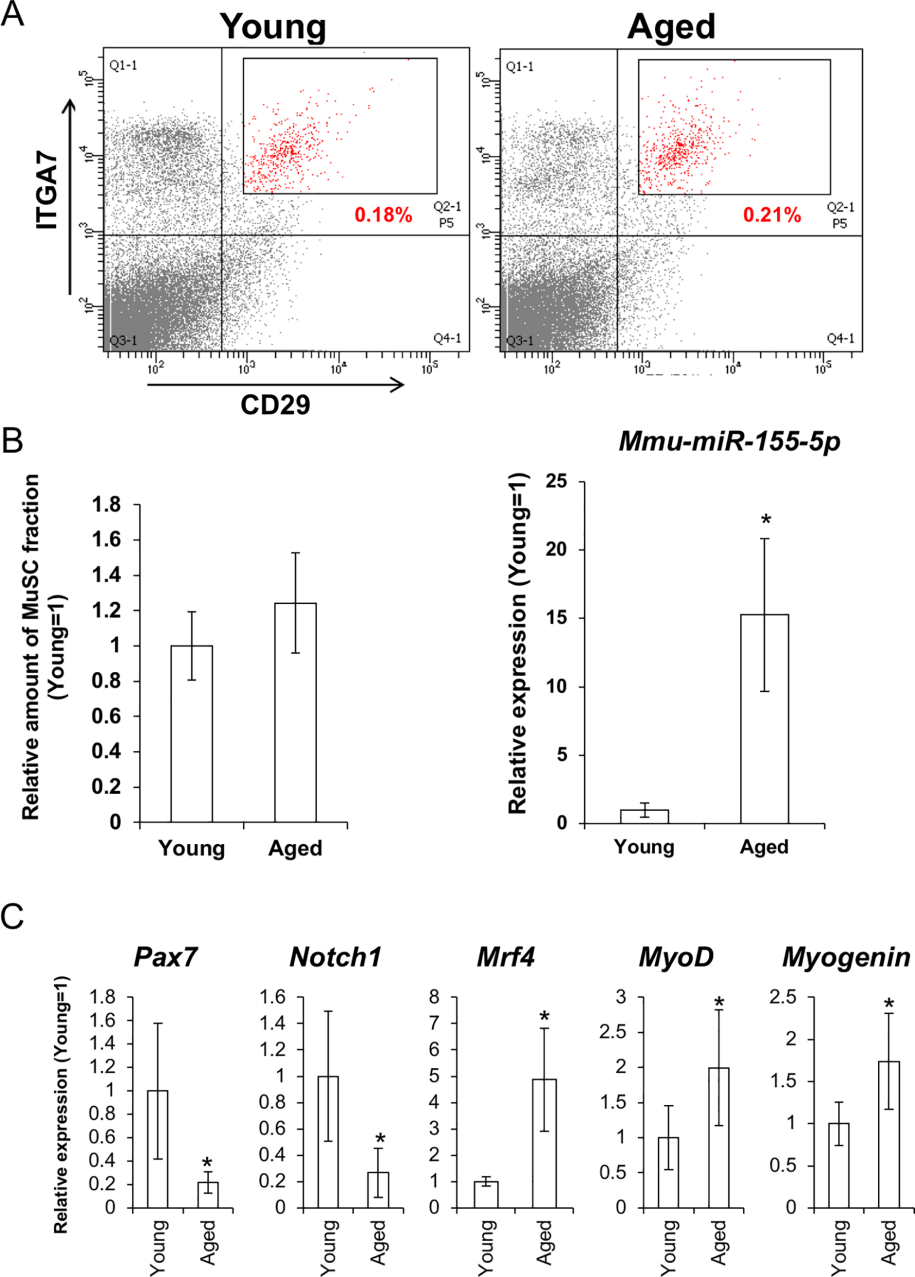
MuSCs of aged muscles showed upregulation of *mmu-miR-155-5p* and differentiating phenotype. (A) FACS sorting of CD11b^negative(neg)^/ CD31^negative^/ CD45^negative^/ITGA7^positive^/CD29^positive^ fractions including MuSCs from young and aged muscles. (B) relative cell numbers of the MuSC in aged muscles to that of young mice (left). Right figure shows relative expression level of *mmu-miR-155-5p* in the MuSCs of aged muscles (N = 6 each). Asterisk indicates significant difference at P < 0.05. (C) gene expressions of undifferentiated (*Pax7* and *Notch1*) and differentiating markers (*Mrf4*, *MyoD*, and *Myogenin*) in the young and the aged MuSCs (N = 6 each).

Existing rates of the CD11b^neg^ /CD 31^neg^ /CD45^neg^/ITGA7^pos^/CD29^pos^ did not change between young and aged mice (Fig 1A and 1B).

The expression level of *miR-155-5p* was 15-fold higher in aged MuSCs. Also in other populations, upregulation of miR-155 occurred (S1 Fig).

When examining gene expression in the population containing MuSCs, undifferentiated MuSC markers *Pax7* and *Notch1* were downregulated in the MuSCs of aged mice. In contrast, genes indicating differentiation committed status, such as *Mrf4*, *MyoD*, and *Myogenin*, were upregulated in the MuSCs of aged mice.

### *miR-155* activated muscular differentiation in C2C12 cells

**Fig 2:**
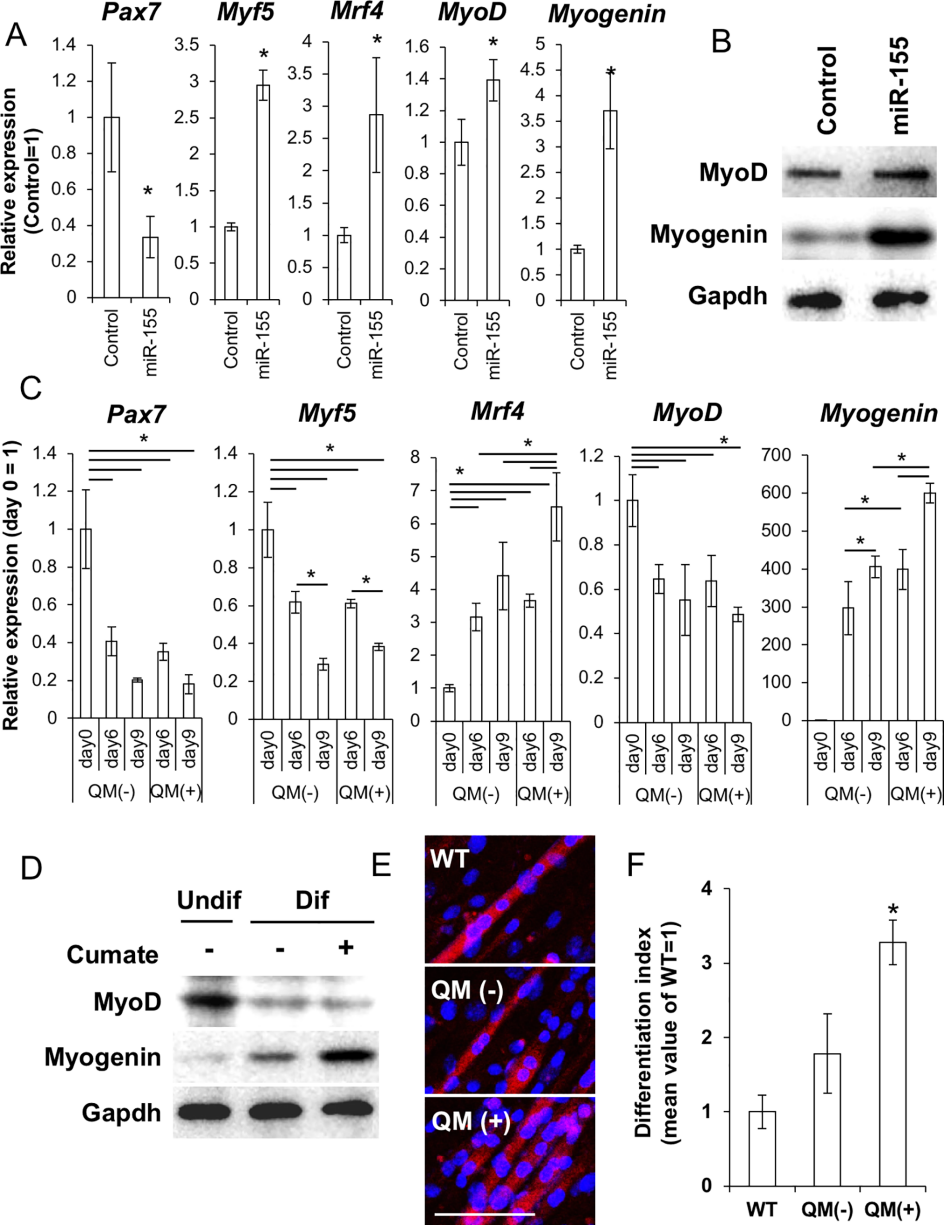
Overexpression of mmu-miR-155 upregulated differentiation associated genes in C2C12 cells. (A) qPCR analysis for undifferentiated markers (Pax7 and Myf5) and differentiated markers (Mrf4, MyoD, and Myogenin) in mmu-miR-155 overexpressing C2C12 cells at 48 h of transfection (N = 3). Asterisks indicate significant differences at P < 0.05 between control transfected with EGFP control plasmid. (B) WB analysis of the differentiated markers MyoD and Myogenin in mmu-miR-155 overexpressing C2C12 cells at 48 h of transfection. (C) effect of the cumate-induced mmu-miR-155 expression on the marker gene expression in C2C12 cells during differentiation. QM is abbreviation of cumate treatment (N = 3). Asterisks indicate significant differences at P < 0.05. (D) WB analysis for differentiated markers MyoD and Myogenin at day 0 and day 9 of differentiation. (E) immunofluorescence for wildtype (WT) C2C12 cells, C2C12 cells containing the cumate-inducible mmu-miR-155 system (QM-mmu-miR-155) with and without cumate at day 12 after differentiation induction. Scale bars = 100 μm. (F) Myotube formation efficiency at day 12 after differentiation induction with or without cumate. Y-axis of the right figure shows relative differentiation index value to differentiated non-transgenic C2C12 cells (differentiation index value of undifferentiated cells = 0, N = 6). Asterisk means significant difference at P < 0.05.

We hypothesized that miR-155 is responsible for activating MuSCs in aged muscles. To investigate this, we overexpressed *miR-155* using the miExpress EGFP-*mmu-miR-155* plasmid in C2C12 cells under normal culture condition with 10%FCS-DMEM. When gene expression was observed on day 2 after transfection, *Pax7* expression was decreased. In contrast, the differentiation markers *Myf5*, *Mrf4*, *MyoD*, and *Myogenin* were upregulated in the miR-155-expressing cells (Fig 2A).

Upregulation of the differentiation markers MyoD and Myogenin was confirmed by western blot (WB) analysis (Fig 2B).

We then observed the long-term effect of miR-155 during myogenic differentiation by using a cumate-inducible expression system. Upon induction of *mmu*-*miR-155* expression with cumate, the proliferation rates of C2C12 cells were decreased (S2 Fig).

In the miR-155 expressing C2C12 cells on day 9 of differentiation, expression of *Mrf4* and *Myogenin* increased (Fig 2C).

Myogenin protein expression was also upregulated by miR-155 induction as determined by WB (Fig 2D).

In the cumate-treated cells, differentiated index obtained by normalized nuclear number within myosin heavy chain (MHC)+ myotube indicates that the rate of fused myoblasts [26] was significantly higher than control cells when observed on day 12 of differentiation (Fig 2E and 2F).

On the other hand, miR-155 induction after day 10 of differentiation did not affect myotube formation efficiency (S3 Fig).

These data suggested that miR-155 supports activation of myogenic differentiation, not myogenic maturation, in C2C12 cells.

### *miR-155-5p* expression and myogenesis were activated in the muscle injury model mice

**Fig 3:**
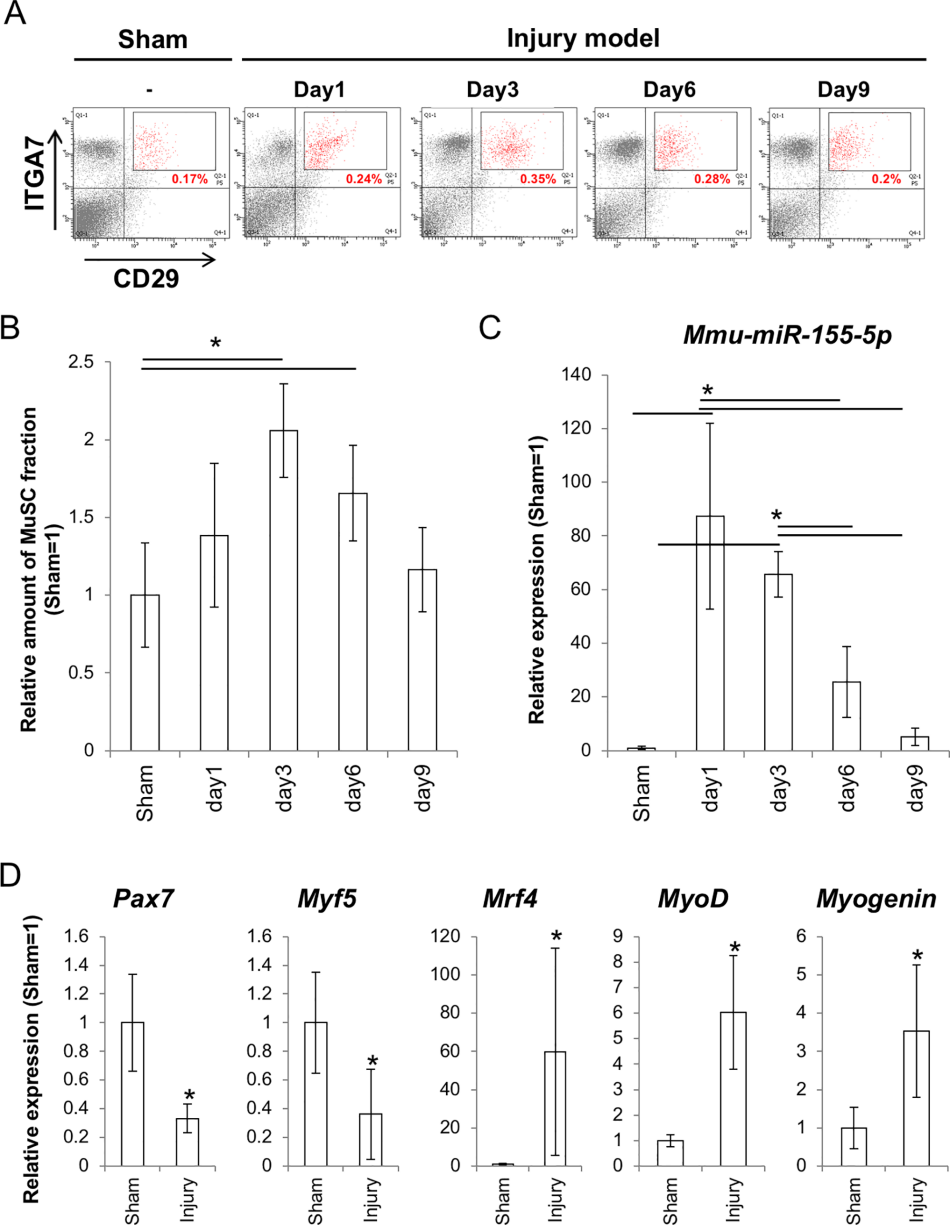
Changes in numbers and in *mmu-miR-155* expression of the cell fraction including MuSCs/ progenitors after injury. (A) FACS analysis for the ITGA7/ CD29 double positive cells after narrowing CD11b^negative(neg)^/ CD31^negative^/ CD45^negative^ populations in injured muscles. Percentages in the figures are means of six independent replicates. (B) relative values of the CD11b^negative(neg)^/ CD31^negative^/ CD45^negative^/ITGA7^positive^/CD29^positive^ fractions to the sham control (N = 6). Sham control indicates samples collected from mice that underwent surgery without damage to the muscular tissues. Statistically significant differences obtained at P < 0.05 between groups are denoted by asterisks. (C) expression changes in *mmu-miR-155-5p* after muscle injury (N = 6). Statistically significant differences obtained between groups are denoted with asterisks. (D) expression change in the marker genes for undifferentiated and differentiated status (N = 6). Asterisks indicate significant differences at P < 0.05 compared to cells from the sham control.

We next observed the expression of *miR-155-5p* and its role *in vivo* using a mouse model of muscle injury. After injury, numbers of the MuSCs increased until day 3 after injury, then returned to normal levels on day 9 (Fig 3A and 3B).

The *miR-155-5p* expression level was dramatically upregulated 80–100 times compared to the baseline level of the sham control when observed at 24 h (day 1) after injury. Then, the upregulated *miR-155-5p* was gradually decreased to the baseline level by day 9 (Fig 3C).

In the MuSCs on day 3 after injury, the undifferentiated and proliferating MuSC markers *Pax7* and *Myf5* were downregulated but differentiation markers *MyoD*, *Myogenin*, and *Mrf4* were upregulated (Fig 3D).

### C/ebpβ inhibited myogenic differentiation and its expression was suppressed by *miR-155*

**Fig 4:**
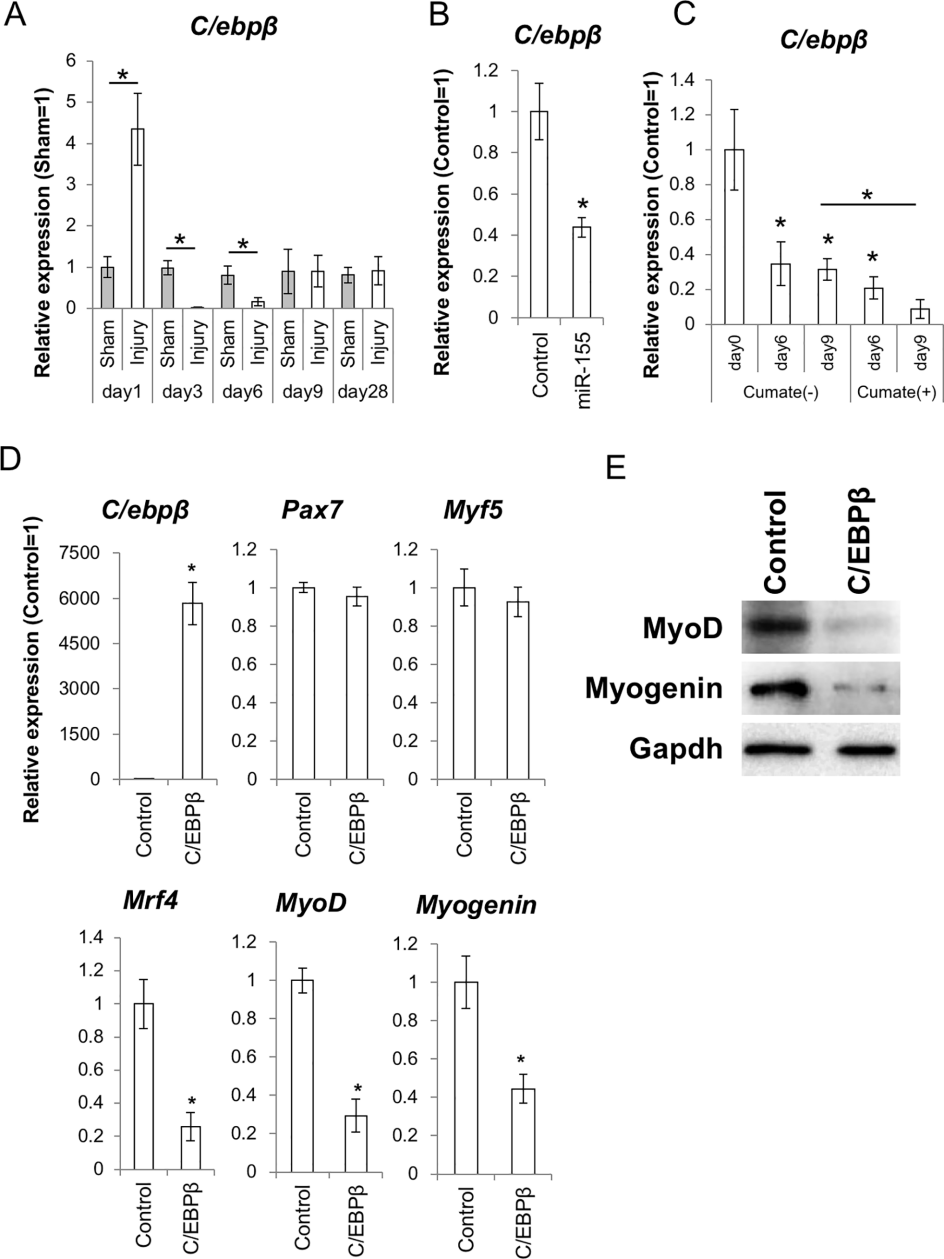
Expression of C/ebpβ in the injury model and in the *mmu-miR-155-* overexpressing cells, and effect of C/ebpβ expression on myogenic differentiation in C2C12 cells. (A) expression level of *C/ebp*β mRNA in the injury model. (B) expression level of *C/ebp*β mRNA in the *mmu-miR-155* overexpressing C2C12 cells (N = 3). (C) expression level of *C/ebp*β mRNA in the cumate-treated C2C12 cells containing the QM-*mmu-miR-155* system (right). Expression of exogenous miR-155 was induced by cumate addition (N = 3). Asterisks indicate significant differences compared to the sham control. (D) effect of C/ebpβ overexpression on the differentiation of C2C12 cells. Samples were collected at day 2 after differentiation induction (N = 3). Asterisks indicate significant differences at P < 0.05 compared to the control cells containing an empty plasmid. (E) WB analysis for the differentiated markers MyoD, Myogenin, and Mrf4 in the C/ebpβ overexpressing C2C12 cells at day 2 of differentiation induction.

To determine the mechanism by which miR-155 activates myogenic differentiation, we focused on the role of C/ebpβ. Until now, it has been well demonstrated that C/ebpβ is a direct target of *miR-155-5p* both in mice and humans [18, 20]. Meanwhile, there is some evidence that C/ebpβ is an essential molecule for maintaining the undifferentiated state of MuSCs [27]. In the injury model used in this study, the expression level of *C/ebp*β was upregulated at day 1 after operation, probably because of many acute inflammatory reactions such as elevated cytokine expression. Then, *C/ebp*β expression was significantly suppressed from day 3 to day 6 in injured muscles (Fig 4A). In the *mmu-miR-155*-overexpressing C2C12 cells, *C/ebp*β expression was downregulated (Fig 4B).

Attenuation of *C/ebp*β by miR-155 was also confirmed in cumate-activated cells (Fig 4C).

On the other hand, overexpression of C/ebpβ in C2C12 cells induced downregulation of the differentiation markers *MyoD*, *Myogenin*, and *Mrf4* (Fig 4D and 4E) in differentiating C2C12 cells at day 3 of differentiation induction. From these results, we concluded that miR-155 supports muscular differentiation through suppressing C/ebpβ, which maintains the undifferentiated status of MuSCs.

### Injury-induced myogenic differentiation was inhibited in *miR-155* knockout cells

**Fig 5:**
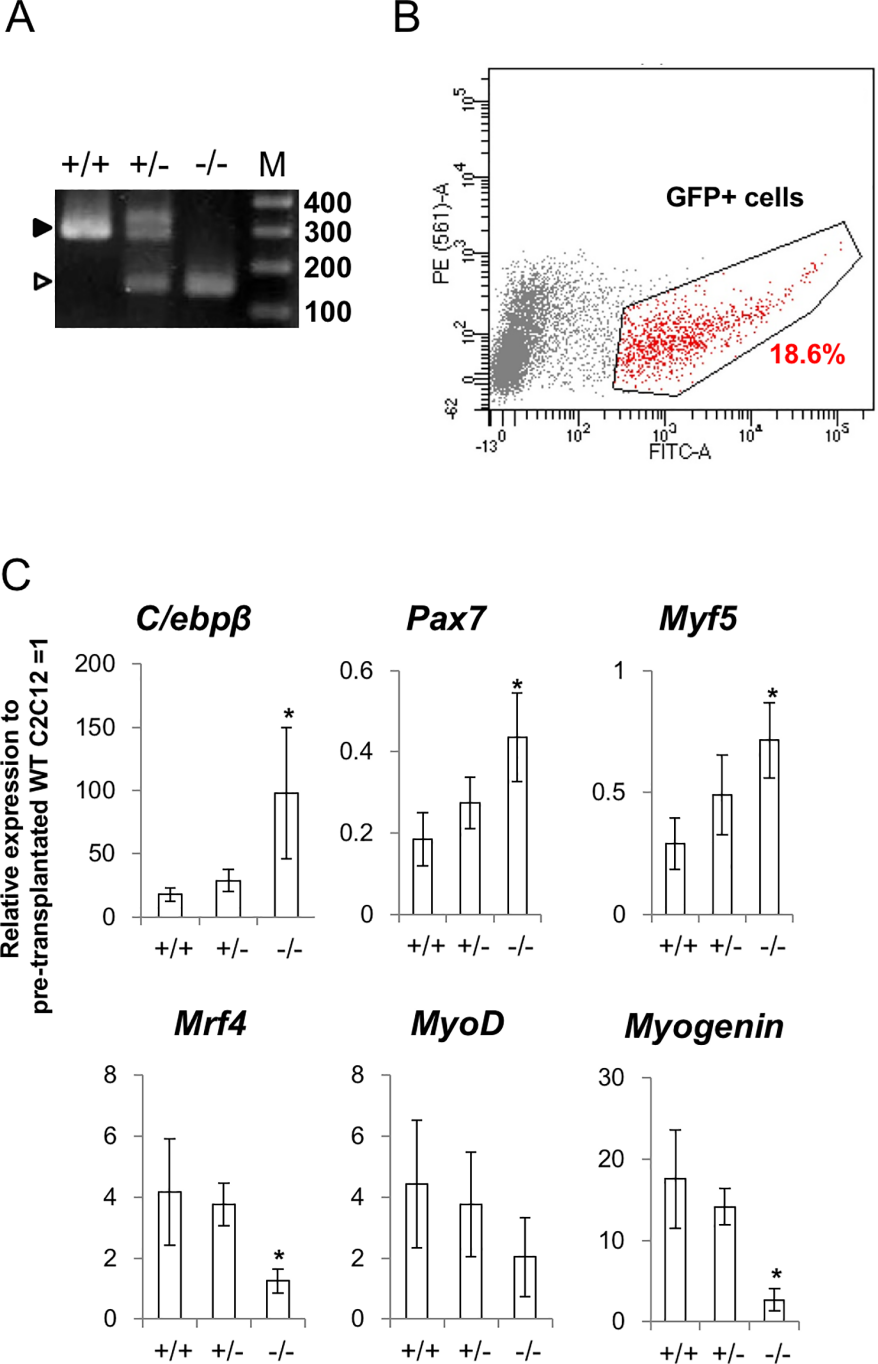
Cell transplantation assay in the injury model using a *miR-155* knockout C2C12 cell-line. (A) Genomic PCR represents shortening of the *mmu-miR-155* gene locus. Black arrow indicates the PCR amplicon size from the WT genome and the white arrow indicates that from the knocked-out (KO) cells. (B) FACS of the GFP-labeled KO C2C12 cells. We sorted the fraction in the black lines (red dots) for this study. (C) gene expression changes of *C/ebp*β, undifferentiated cell markers *Pax7* and *Myf5*, and differentiated markers *MyoD*, *Myogenin*, and *Mrf4*. +/+ means wildtype C2C12 cells, +/- means heterozygous of miR-155 KO C2C12 cells, and -/- means null mutant of miR-155 KO C2C12 cells (N = 6). Asterisks indicate significant differences at P < 0.05 between each group.

We then examined whether miR-155 was important for injury-induced myogenic differentiation using a miR-155-deleted C2C12 cell line. To prepare the miR-155 deleted cell line, we performed CRISPR-Cas9-mediated gene deletion using two guide RNAs [20]. We produced one cell line lacking the *miR-155* gene (Fig 5A).

The miR-155-deleted C2C12 cells showed a normal phenotype with respect to cell morphology, proliferation, and *in vitro* differentiation (S4 Fig).

The cell line was labeled with GFP by introducing the piggyback-CAG-GFP plasmid, and then transplanted to injured C3H mice, which have the same genetic background as the C2C12 cell line. Three days after transplantation, the normal C2C12 cells showed decreased expression of *C/ebp*β and *Pax7*, and upregulation of *MyoD*, *Myogenin*, and *Mrf4* compared with the levels of pre-transplantation C2C12 cells. In contrast, both the downregulation of *C/ebp*β and *Pax7* and the upregulation of *MyoD*, *Myogenin*, and *Mrf4* were altered in the miR-155 KO C2C12 cells (Fig 5C).

### Notch1 bound to the promoter region of *miR-155* host gene and repressed expression of *miR-155*

**Fig 6:**
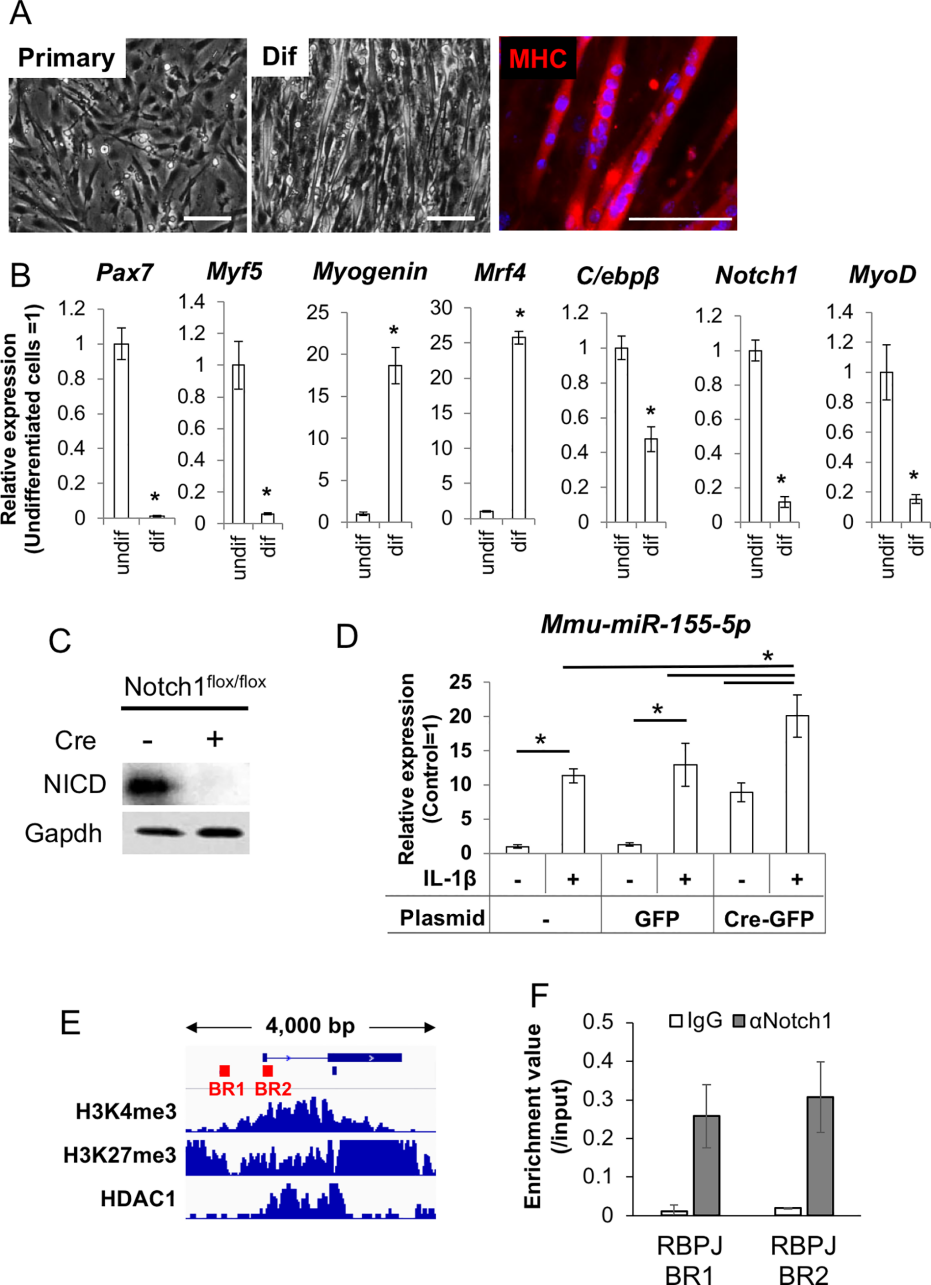
Notch1 suppress expression of *mmu-miR-155* in the MuSCs. (A) primary culture of the mouse MuSCs. Undifferentiated state (Primary), differentiated sate (Dif) and immunofluorescent image with anti-MHC antibody (MHC) are shown. Scale bars = 100 μm. (B) qPCR representing stem cell properties for myogenic differentiation of the cultured MuSCs. Undifferentiated cells (undif) and differentiated cells at day 6 after differentiation induction (dif) were compared. Asterisks indicate significant differences at P < 0.05 between each group. (C) WB analysis to show floxing of Notch1 gene alleles by transfecting a Cre-GFP plasmid. (D) expression levels of *mmu-miR-155-5p* in the Notch1 floxed MuSCs without or with IL1β stimulation. GFP means control determining effect of transfection. (E) promoter region of miR-155 host gene (Mir155HG) and binding peeks of H3K4me3, H3K27me3, and HDAC1. Bars show positions of consensus sequence of Rbpj, which is a partner protein of Notch1 for DNA binding. (F) ChIP-qPCR with Notch1 antibody for the Notch1/Rbpj.

From the above results, we concluded that the increased expression of miR-155 could be an important factor for activating differentiation of the MuSCs both *in vitro* and *in vivo*. Then, we aimed to identify an aging-specific mechanism that leads to miR-155 upregulation. Some studies showed decreased Notch1 signaling in aged MuSCs [7, 8, 28]. Thus, we examined whether Notch1 acts as a repressor for miR-155 in MuSCs. To perform this *in vitro* experiment, we prepared primary cultures including MuSCs/myoblasts from Notch1 conditional KO (Notch1^flox/flox^) mice (Fig 6A).

In the primary culture, expression of *Pax7* and *Notch1* were maintained at high levels, but the expression of differentiation markers *MyoD*, *Myogenin*, and *Mrf4* was low. Upon inducing differentiation, expression of *Pax7* was repressed and the differentiation markers were significantly upregulated (Fig 6B).

To observe the effect of *Notch1* deletion, we induced excision of *Notch1*^*flox/flox*^ genes by transiently transfecting a Cre-GFP plasmid and purified the KO cells by FACS using GFP fluorescence. WB for the Notch1-intracellular domain (NICD) showed that NICD expression was undetectable in the GFP+ fractions (Fig 6C).

In the *Notch1*-deleted cells, *miR-155-5p* expression was dramatically increased even under normal culture conditions. When the Notch1-deleted cells were stimulated with IL-1β, upregulation of *miR-155-5p* was enhanced compared with the upregulation observed in wild type cells (Fig 6D).

To determine whether Notch1 can bind to the promoter region of the *miR-155 host gene (miR-155HG)* in muscular lineage cells, we performed ChIP analysis. Target regions for ChIP-PCR were designed based on previous papers [29] and information from public databases. Two consensus sequences of Rbpj (TGRGGA), which binds to Notch1 before binding with DNA, are found at approximately -2 kbp and +100 bp from the TSS of the miR-155 host gene (Fig 6E).

Especially the +4-+116 bp site (binding region 2) corresponded with the binding peak of inactivated histone marks H3K4me3, H3K27me3, and Hdac1 when analyzed in silico (Fig 6F).

These results suggested that Notch1 binds to the promoter region of *miR-155*, likely repressing its transcription in MuSCs/myoblasts. From the above results, it was thought that disrupting Notch1 could be a direct reason for miR-155 upregulation in aged muscles.

## Discussion

Essential roles of miRNA in myogenic differentiation of MuSCs have been clearly demonstrated by studies on knockout mice for Dicer1, which is an RNase III endonuclease that produces mature miRNA [30, 31]. The specific functions of some individual miRNAs on cell proliferation and differentiation in myogenic processes have also been elucidated. For example, miR-1, miR-133, and miR-206 are involved in regulating myoblast proliferation and differentiation [32, 33]. Crist *et al*. demonstrated that miR-27a targets paired box protein 3 (Pax3), which is an important transcription factor for MuSC quiescence [34]. Recently, Zhai *et al*. discovered that miR-217 is involved in inducing MuSC differentiation [35]. In this study, we focused on miR-155, which is an inflammation- and aging-associated miRNA [20–22].

In the MuSCs of aged muscles, rates of the CD11b^neg^/CD31^neg^/CD45^neg^/ITGA7^pos^/CD29^pos^ fraction, in which MuSCs are concentrated, did not change between young and aged mice. Some previous studies shown a reduction in MuSC numbers associated with aging [36, 37]. In contrast, many studies have also reported no change in the number of MuSCs in aged muscle [8, 38]. This discrepancy may be due to the methods and antibodies used for measuring MuSCs. For example, Shefer G *et al*. quantified the numbers of satellite cells by fluorescent microscopy with anti-Pax7 and anti-MyoD antibodies [36]. Verdijk *et al*. also counted the number of MuSCs by immunofluorescence after discriminating between type I and type II fibers [37]. In the present study, we performed FACS with cell surface markers that are present on quiescent and activated satellite cells, and also early myoblasts could be positive. This indicates that our approach should produce a mixture of these cells, thus we were not able to detect a decline in MuSC population numbers. In the FACS sorted MuSC population, expression level of *mmu-miR-155-5p* was clearly upregulated. In aged tissues including muscles, aging-associated inflammation is a well-established phenomenon [39]. It has also been well demonstrated that proinflammatory stimuli upregulate miR-155 [20, 40, 41]. Thus, it is not surprising that increased expression of miR-155 was detected in aged muscles.

*In vitro* overexpression experiments clearly showed that miR-155 activated myogenic differentiation of C2C12 model cells. Regarding the function of miR-155 as a differentiation activator, we recently reported that miR-155 blocks neural stem cell self-renewal by suppressing the expression of transcription factors involved in stemness both in mouse and human cells [18]. On the other hand, Seok *et al*. reported that miR-155 represses myogenic differentiation by targeting Mef2A, a key myogenic transcription factor, in C2C12 cells [42]. This discrepancy may be due to the different experimental models used and different timing for observation. It is possible that the cells in the early phase of differentiation status around day 3 are unstable and various factors such as the amount of miRNA, cell culture conditions, and cell density can disrupt the results. To stabilize the experiment, we analyzed the miR-155 overexpressing cells after purification by FACS using GFP fluorescence that was by-product of miR-155. This method enables exclusion of contamination by the cells with no or low miR-155 expression. Furthermore, to keep the high expression level of miR-155 during *in vitro* differentiation, we used the cumate-inducible *mmu-miR-155* expression system, in which miR-155 overexpression is induced by cumate supplementation. As a result, it was shown that *miR-155* supported myogenic differentiation of C2C12 cells. To confirm the function of miR-155 in myogenesis by a different approach, we conducted an *in vivo* study using a mouse model of muscle injury, expecting that miR-155 would be upregulated by injury-induced inflammation. As expected, *miR-155-5p* was significantly upregulated in the MuSCs of injured animals. *miR-155-5p* expression was especially high at the early periods of injury and was maintained at levels over 20 times higher than those in the sham control until day 6 after injury. We especially focused on day 3 after injury, because it is difficult to analyze samples soon after injury, such as on day 1, due to disturbances such as bleeding, local ischemia, and infections. On day 3, there was clear evidence demonstrating activation of myogenic differentiation was obtained. At this time point, *miR-155-5p* was highly expressed. In contrast, *C/ebp*β, which is an important transcription factor for SC self-renewal, was significantly downregulated. Until now, some researchers, including our group, demonstrated that *C/ebp*β is a direct target of miR-155. In healthy skeletal muscle, *C/ebp*β expression is restricted to Pax7^pos^ MuSCs and supports self-renewal of MuSCs. However, *C/ebp*β expression was suppressed at early period of the regeneration process; this downregulation is required for myogenesis [27, 43]. In our model, a high level of *C/ebp*β expression was observed on day 1 after surgery. This surge of *C/ebp*β expression may indicate the dominance of stem/progenitor cell proliferation in the injured sites, and decreased expression of *C/ebp*β on day 3 and day 6 suggests that the muscles were committed to differentiation during these periods. Regarding the decreased expression of *C/ebp*β observed on day 3, we hypothesized that miR-155 might be involved, and this reaction would be important for activating differentiation in MuSCs. Consistent with our hypothesis, the overexpression of *mmu-miR-155* suppressed *C/ebp*β and affected differentiation in C2C12 cells. Obviously, expression timing and the amounts of related molecules need to be finely tuned for appropriate regeneration. On the contrary, the overexpression of *C/ebp*β inhibited myogenic differentiation in C2C12 cells. Marchildon *et al*. also experimentally demonstrated that forced expression of C/ebpβ blocks myogenesis, representing increased Pax7 and decreased MyoD, Myogenin, and myosin heavy chain (MHC) expression [27]. Thus, the downregulation of C/ebpβ by miR-155 may be a key step in the switch between proliferation and differentiation of MuSCs to induce subsequent tissue regeneration.

To confirm whether elevated miR-155 levels in the *in vivo* MuSCs relate to differentiation activation, we generated a *miR-155* KO C2C12 cell line and transplanted it in the injured muscles of mice in order to model an *in vivo* myoblast lacking miR-155. As expected, myogenic differentiation was inhibited in the KO cells compared with the differentiation of heterozygous and wild-type cells, indicating that miR-155 is involved in activating differentiation during the muscle regeneration process. Recently, Nie *et al*. analyzed the phenotype of miR-155 KO mice with respect to muscle regeneration and reported that muscle regeneration was delayed in the miR-155 KO mice. They found that the newly generated muscle fibers of miR-155 KO mice are smaller than wild-type fibers at both 14 and 21 days after cardiotoxin injection [44]. Our results are highly consistent with the phenotypes they observed in the endogenous MuSCs. However, in their study, miR-155 was not detected in the primary culture of the MuSCs. This may have occurred because miRNA expression is easily affected by environmental stimulation both *in vivo* and *in vitro*, and therefore, in this case, expression level of miR-155 was abolished through ex vivo culture. Thus, we observed the miR-155 expression in MuSCs directly isolated from tissues by FACS. By this method, the actual expression levels of miR-155 in tissues could be detected. Furthermore, our transplantation study supported the hypothesis that miR-155 is involved in myogenic differentiation *in vivo*.

As a role of miR-155, we focused on differentiation activation of MuSCs through regulation of C/ebpβ. Meanwhile, important functions of C/ebpβ in stem cell survival have been demonstrated [45]. Buck *et al*. showed that C/EBPβ directly inhibits caspase activity, and regulates p53 activity and expression, both of which could relate to cellular viability [46]. These notions suggest that overexpression of miR-155 and excessive downregulation of C/ebpβ could possibly lead to stem cell dysfunction through multiple pathways.

Finally, we looked for a specific cue triggering upregulation of miR-155 in the aged MuSCs. In the muscular tissues of aged animals, decreased Notch1 signaling, an important regulator for various genes, was frequently observed [7, 8, 47]. Thus, we hypothesized that defective Notch1 signaling may be a reason for the increased level of miR-155 in the aged MuSCs. As expected, significant upregulation of miR-155 was observed during Notch1 deletion experiments. ChIP analysis showed that Notch1 binds to the promoter region of miR-155 and may be involved in repressing miR-155 in MuSCs. Consistent with our observation, Wang *et al*. reported that Notch signaling represses miR-155 expression through Rbpj, which is a partner for DNA binding of Notch1, and loss of Notch/Rbpj signaling upregulates miR-155 in bone marrow endothelial cells [29]. From these results, it was thought that Notch1 maintains robustness against inflammation associated tissue degenerations, and the reduction of Notch1 expression, a well-known characteristic in aged muscles, could result in exceeding expression of its target genes including miR-155. Consistent with this idea, the Notch1 KO MuSCs expressed higher level of miR-155 in response to IL-1β supplementation compared with WT cells. This result could mean that Notch1 is important not only for suppressing miR-155 expression in normal conditions, but also for maintaining an appropriate level of miR-155 expression. However, in aged muscles, chronic inflammation and Notch1 deficiency occur simultaneously. Subsequently, excessive expression of miR-155 is induced, and as a result, an imbalance between maintenance and commitment occurs in the stem cell pool.

## Conclusions

Here, we demonstrated that overexpression of miR-155 triggered by both inflammation and loss of Notch1, regulates activation of MuSC differentiation by suppressing *C/ebp*β. This may be a mechanism that explains some of the aging-associated changes which occur in muscles. With advancement in technology for administration of oligo-RNAs, miRNA therapies become a real option for the management of various diseases [48]. Therapeutics using miR-155 inhibitors have also been developed for lung cancer [49]. Meanwhile, some studies suggest tissue protective roles of miR-155 in injured tissues [50, 51]. Clearly, spatiotemporal-specific regulation is crucial to attaining effective therapeutic intervention against miR-155. To do so, a better understanding of the detailed biological functions, importance, and appropriate quantities of miR-155 for tissue regeneration would be essential.

## Supporting information

S1 Fig Expression levels of miR-155 (pre-mature form) in the ITGA7^pos^/CD29^pos^, ITGA7^pos^/CD29^neg^, ITGA7^neg^/CD29^pos^, and ITGA7^neg^/CD29^neg^ population. These populations were specified after exclusion of CD11b^neg^/CD31^neg^/CD45^neg^ fractions.
(TIFF)

S2 Fig Effect of miR-155 expression on cell proliferation of C2C12 cells. Asterisks indicate significant differences at P < 0.05 compared with the cumate untreated control (N = 3).
(TIFF)

S3 Fig Effect of miR-155 on the myogenic fiber formation. Cumate treatments were performed for 48 hours from 10 days after differentiation induction.
(TIFF)

S4 Fig Phenotype observation of the miR-155 knockout C2C12 cells. Upper figure shows cell proliferation of the wildtype, heterozygous and null mutant cells in 10%FCS-DMEM (normal culture) and 2%HS-DMEM (differentiation). Lower figures show gene expressions of myogenic markers in the wildtype, heterozygous and null mutant C2C12 cells.
(TIFF)
