# Supplementary figures and images for: Inflammation-associated miR-155 activates differentiation of muscular satellite cells
Source: PLoS One. 2018 Oct 1;13(10):e0204860. doi: 10.1371/journal.pone.0204860 (PMC6166968; doi:10.1371/journal.pone.0204860)

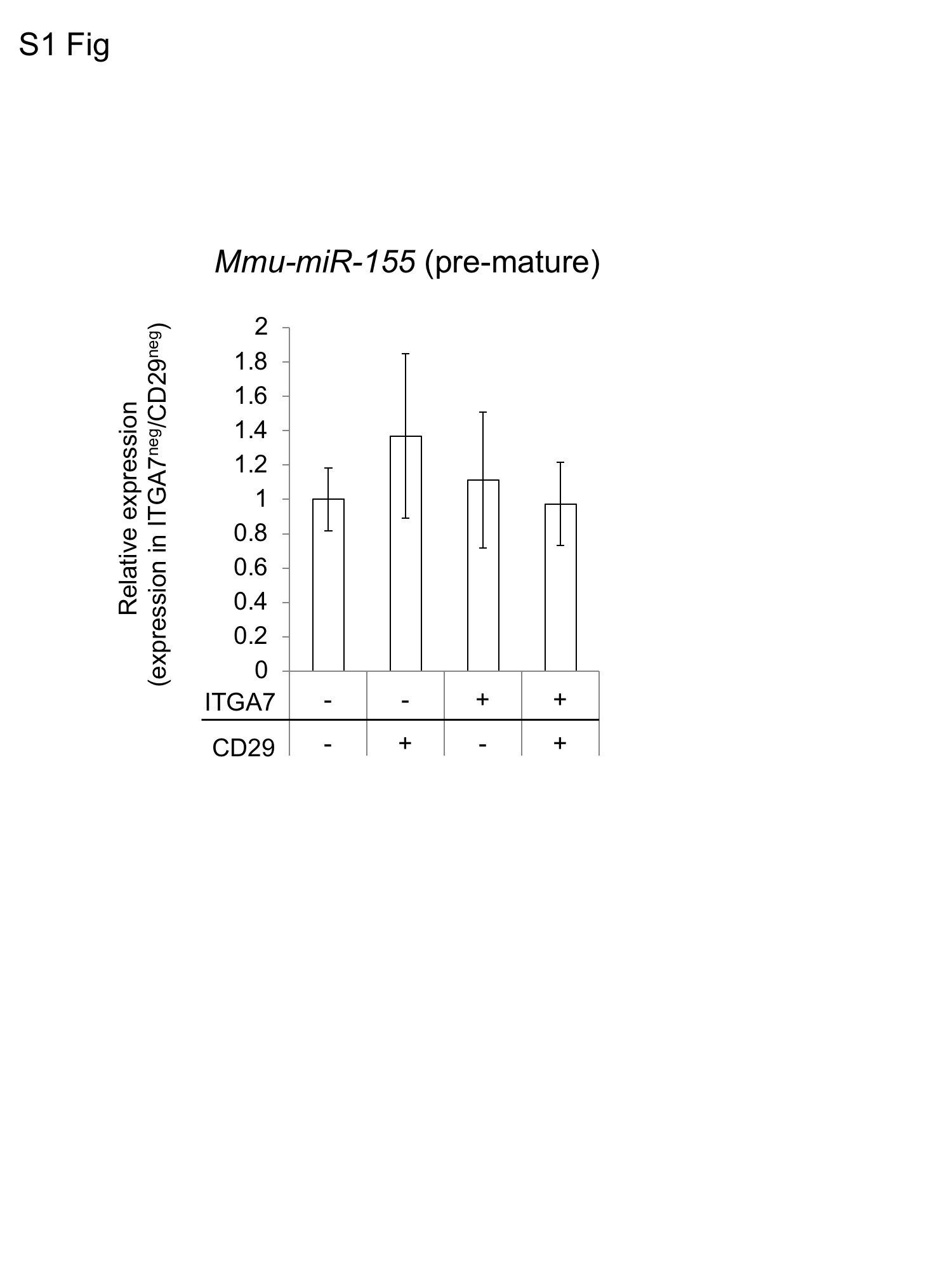

Supplement: S1 Fig — These populations were specified after exclusion of CD11bneg/CD31neg/CD45neg fractions. (TIFF) [file pone.0204860.s001.tiff]

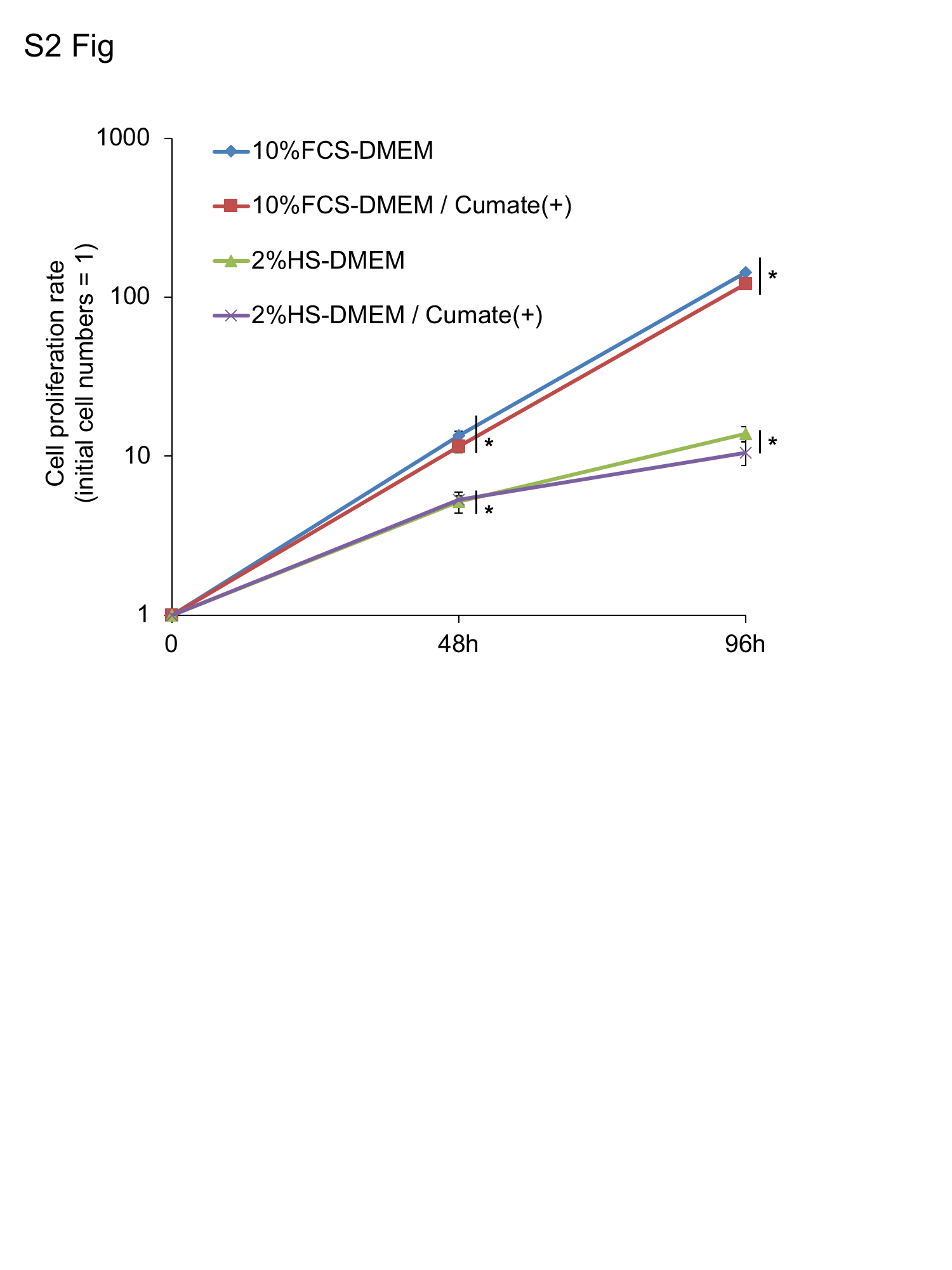

Supplement: S2 Fig — Asterisks indicate significant differences at P < 0.05 compared with the cumate untreated control (N = 3). (TIFF) [file pone.0204860.s002.tiff]

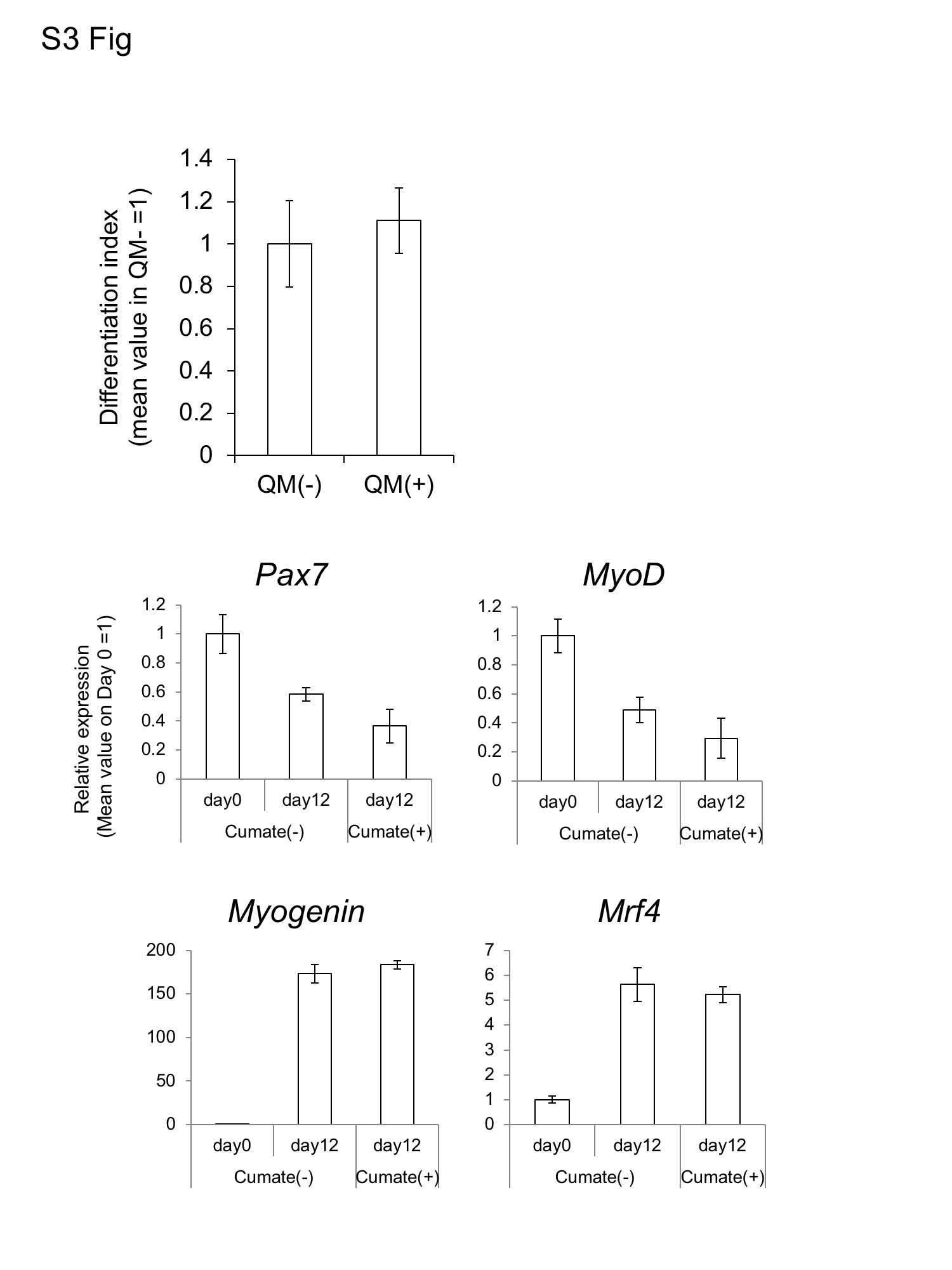

Supplement: S3 Fig — Cumate treatments were performed for 48 hours from 10 days after differentiation induction. (TIFF) [file pone.0204860.s003.tiff]

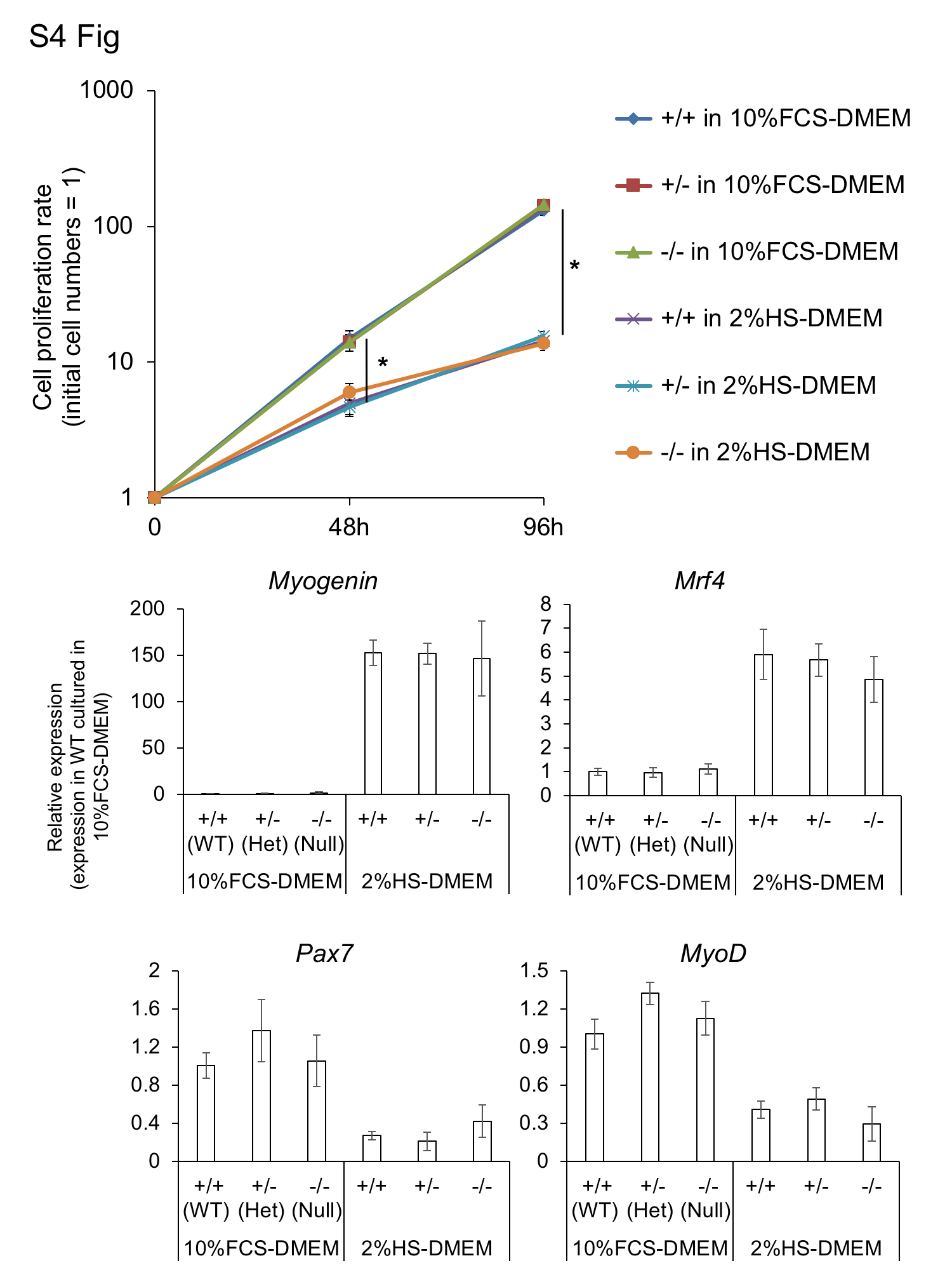

Supplement: S4 Fig — Upper figure shows cell proliferation of the wildtype, heterozygous and null mutant cells in 10%FCS-DMEM (normal culture) and 2%HS-DMEM (differentiation). Lower figures show gene expressions of myogenic markers in the wildtype, heterozygous and null mutant C2C12 cells. (TIFF) [file pone.0204860.s004.tiff]
